# Aldehyde dehydrogenase 1A1 increases NADH levels and promotes tumor growth via glutathione/dihydrolipoic acid-dependent NAD^+^ reduction

**DOI:** 10.18632/oncotarget.17688

**Published:** 2017-05-08

**Authors:** Baiyun Wang, Xue Chen, Zixi Wang, Wei Xiong, Tao Xu, Xinyuan Zhao, Yang Cao, Yanru Guo, Lin Li, She Chen, Song Huang, Xiaodong Wang, Min Fang, Zhirong Shen

**Affiliations:** ^1^ Peking University-Tsinghua University-National Institute of Biological Sciences Joint Graduate Program, School of Life Sciences, Tsinghua University, Beijing, 100084, China; ^2^ Joint Center for Life Sciences, and School of Life Sciences, Peking University, Beijing, 100871, China; ^3^ National Institute of Biological Sciences, Beijing, 102206, China

**Keywords:** aldehyde dehydrogenase, lung cancer, glutathione, dihydrolipoic acid, NAD^+^/NADH ratio

## Abstract

Aldehyde dehydrogenase 1A1 (ALDH1A1) is a member of the aldehyde dehydrogenase superfamily that oxidizes aldehydes to their corresponding acids, reactions that are coupled to the reduction of NAD^+^ to NADH. We report here that ALDH1A1 can also use glutathione (GSH) and dihydrolipoic acid (DHLA) as electron donors to reduce NAD^+^ to NADH. The GSH/DHLA-dependent NAD^+^-reduction activity of ALDH1A1 is not affected by the aldehyde dehydrogenase inhibitor or by mutation of the residues in its aldehyde-binding pocket. It is thus a distinct biochemical reaction from the classic aldehyde-dehydrogenase activity catalyzed by ALDH1A1. We also found that the ectopic expression of ALDH1A1 decreased the intracellular NAD^+^/NADH ratio, while knockout of ALDH1A1 increased the NAD^+^/NADH ratio. Simultaneous knockout of ALDH1A1 and its isozyme ALDH3A1 in lung cancer cell line NCI-H460 inhibited tumor growth in a xenograft model. Moreover, the ALDH1A1 mutants that retained their GSH/DHLA-dependent NAD^+^ reduction activity but lost their aldehyde-dehydrogenase activity were able to decrease the NAD^+^/NADH ratio and to rescue the impaired growth of ALDH1A1/3A1 double knockout tumor cells. Collectively, these results suggest that this newly characterized GSH/DHLA-dependent NAD^+^-reduction activity of ALDH1A1 can decrease cellular NAD^+^/NADH ratio and promote tumor growth.

## INTRODUCTION

The aldehyde dehydrogenase superfamily in humans is comprised of 19 isozymes that are responsible for the oxidation of endogenous and exogenous aldehydes. ALDH1A1 can catalyze the irreversible oxidation of retinal to retinoic acid, which is required for growth and development, and processes anti-cancer activities [1–3]. Other substrates of ALDH1A1 include acetaldehyde, 3,4-dihydroxyphenylacetaldehyde, and so on [4–6]. Another aldehyde dehydrogenase, ALDH3A1, functions in the detoxification of lipid peroxidation-derived reactive aldehydes like 4-hydroxynonenal [7]. Increased levels of ALDH1A1 and ALDH3A1 in various human cancers have been associated with enhanced tumorigenic ability, chemo-resistance, and poor prognosis [8–10]. ALDH1A1 and ALDH3A1 can metabolize the active form of oxazaphosphorine drugs (e.g., cyclophosphamide, mafosfamide, and ifosfamide) and are, at least partially, responsible for the chemo-resistance associated with these drugs [11, 12]. However, it is still not clear how aldehyde dehydrogenases contribute to the increased tumorigenesis and the poor prognosis.

Nicotinamide adenine dinucleotide (NAD^+^) is a ubiquitous coenzyme. NAD^+^ can accept electrons and can be reduced to NADH in many redox reactions [13]. The NAD^+^/NADH ratio reflects cellular redox status and is important in maintaining redox homeostasis [14, 15]. The NAD^+^/NADH ratio also influences cellular metabolism by affecting the activity of NAD^+^-dependent enzymes, including deacetylase sirtuins and dehydrogenases like lactate dehydrogenase, pyruvate dehydrogenase, and isocitrate dehydrogenase [16, 17]. Recently, Denis *et al*. showed that increase of the NAD^+^/NADH ratio ameliorated the proliferative and metabolic defects caused by an impaired electron transport chain [18]. Zhao *et al*. reported that the NAD^+^/NADH ratio is decreased in certain cancer cell lines and showed that compounds that increase the NAD^+^/NADH ratio have specific toxicity towards cancer cells [19]. These findings have reinforced the potential significance of selectively manipulating the NAD^+^/NADH ratio under pathological settings to achieve therapeutic effects.

We report here that ALDH1A1/3A1 can catalyze a previously-unreported enzymatic reaction. In addition to aldehydes, we discovered that these enzymes can use glutathione (GSH) and dihydrolipoic acid (DHLA) as the electron donor to reduce NAD^+^ to NADH. Moreover, using ALDH1A1 mutants that retained this GSH/DHLA-dependent NAD^+^-reduction activity but lost the aldehyde dehydrogenase activity, we found that ALDH1A1 can decrease the cellular NAD^+^/NADH ratio and promote tumor growth in xenograft models via the GSH/DHLA-dependent NAD^+^-reduction activity.

## RESULTS

### Identification of a GSH/DHLA-dependent NAD^+^-reduction activity

**Figure 1:**
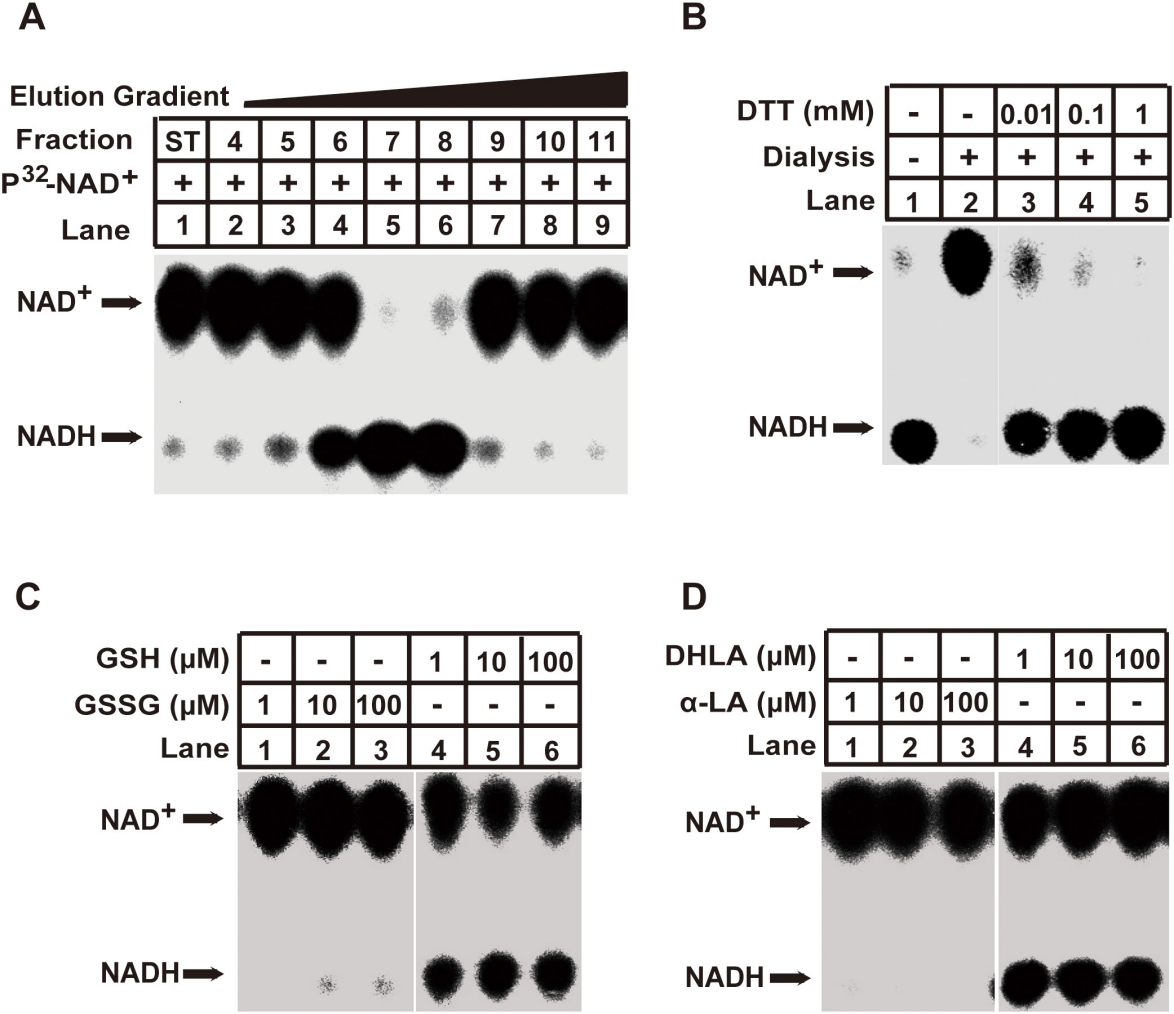
Identification of the GSH/DHLA-dependent NAD^+^-reduction activity in cellular extracts **(A)** 5 mg of cytosolic extracts (S-100) from EKVX cells were fractionated via 1 mL Q Sepharose HP columns with a salt gradient elution from 150 to 550 mM NaCl. 1 mL fractions were collected, and assayed for the NAD^+^-reduction activity as described in the Materials and Methods. Briefly, 3 μL aliquots of the indicated fractions were incubated with P^32^- NAD^+^ at 30°C in a total volume of 30 μL for 1 hour. The reaction was stopped via the addition of trichoroacetic acid, and the supernatants were developed by thin layer chromatography (TLC). The positions of NAD^+^ and NADH are indicated. **(B)** The active fraction (Q7) from panel **(A)** was dialyzed against the buffer lacking dithiothreitol (DTT) at 4°C for 16 hours. NAD^+^ reduction was assayed in the presence of different concentrations of DTT. (**C** and **D**) The NAD^+^ reduction of the dialyzed active fraction (Q7) was visualized in the presence of the indicated concentrations of glutathione (GSH) or oxidized glutathione (GSSG) (C) and dihydrolipoic acid (DHLA) or α-lipoic acid (α-LA) (D).

We set up an *in vitro* assay to explore enzymatic activities related to NAD^+^ metabolism in cancer cells. Cell extracts (S-100, supernatants of cell lysates that had been centrifuged at 100,000 g) from the EKVX lung cancer cell line were fractionated on a Q HP anion exchange column. Individual fractions were then incubated with P^32^-labeled NAD^+^ at 30°C for an hour. Thin layer chromatography (TLC) analysis was then performed. A robust NAD^+^ to NADH reduction activity was detected in column fractions 6 to 8 (Figure 1A). NAD^+^ is typically reduced to NADH in dehydrogenase-catalyzed redox reactions in which a hydride ion (H-) is transferred from an electron donor to NAD^+^. Careful consideration of the buffer composition led to our speculation that the electron donor for the observed NAD^+^ to NADH reduction activity appeared, surprisingly, to be the reducing agents (dithiothreitol, DTT) in the assay buffer. To test this possibility, the active fraction (Q7) was dialyzed against buffer lacking DTT. After dialysis, the Q7 fraction indeed lost its NAD^+^-reduction activity. Adding back DTT restored this activity (Figure 1B). These results suggest that the reducing agent DTT is the electron donor for the observed NAD^+^-reduction.

Given that DTT is an exogenous reducing agent, we next asked whether any endogenous reducing agents could serve as electron donors for the observed NAD^+^-reduction. Glutathione (GSH) and dihydrolipoic acid (DHLA, the reduced form of α-lipoic acid) are widely-employed endogenous reducing agents in metabolism [20]. We found that the Q7 fraction, in the presence of GSH and DHLA, could reduce NAD^+^ to NADH. However, no NAD^+^ reduction activity occurred when the respective oxidized forms of these compounds, GSSG and α-lipoic acid, were present in the assay buffer (Figure 1C and 1D). These results indicate the presence of a candidate dehydrogenase in the Q7 fraction that can catalyze the transfer of electrons from GSH and DHLA to NAD^+^. This enzymatic activity is referred to henceforth as GSH/DHLA-dependent NAD^+^-reduction activity.

To further confirm that the observed GSH/DHLA-dependent NAD^+^-reduction activity was not generated from non-specific effects of reducing agents or dehydrogenases, we made three previously-characterized recombinant dehydrogenases and used them in the same assay; these included glyceraldehyde 3-phosphate dehydrogenase (GAPDH) and two lactate dehydrogenases (LDHA and LDHB). When GAPDH and LDHA/B were assayed using GSH as the electron donor, no NAD^+^-reduction activity was detected. GAPDH and LDHA/B only catalyzed NAD^+^ reduction when their specific substrates (glyceraldehyde 3-phosphate and lactate) were used as the electron donors (Supplementary Figure 1A and 1B). These results showed that the GSH/DHLA-dependent NAD^+^-reduction activity present in fraction Q7 was not shared by other dehydrogenases such as GAPDH and LDHA/B.

### ALDH1A1 catalyzes the observed GSH/DHLA-dependent NAD^+^ reduction

**Figure 2:**
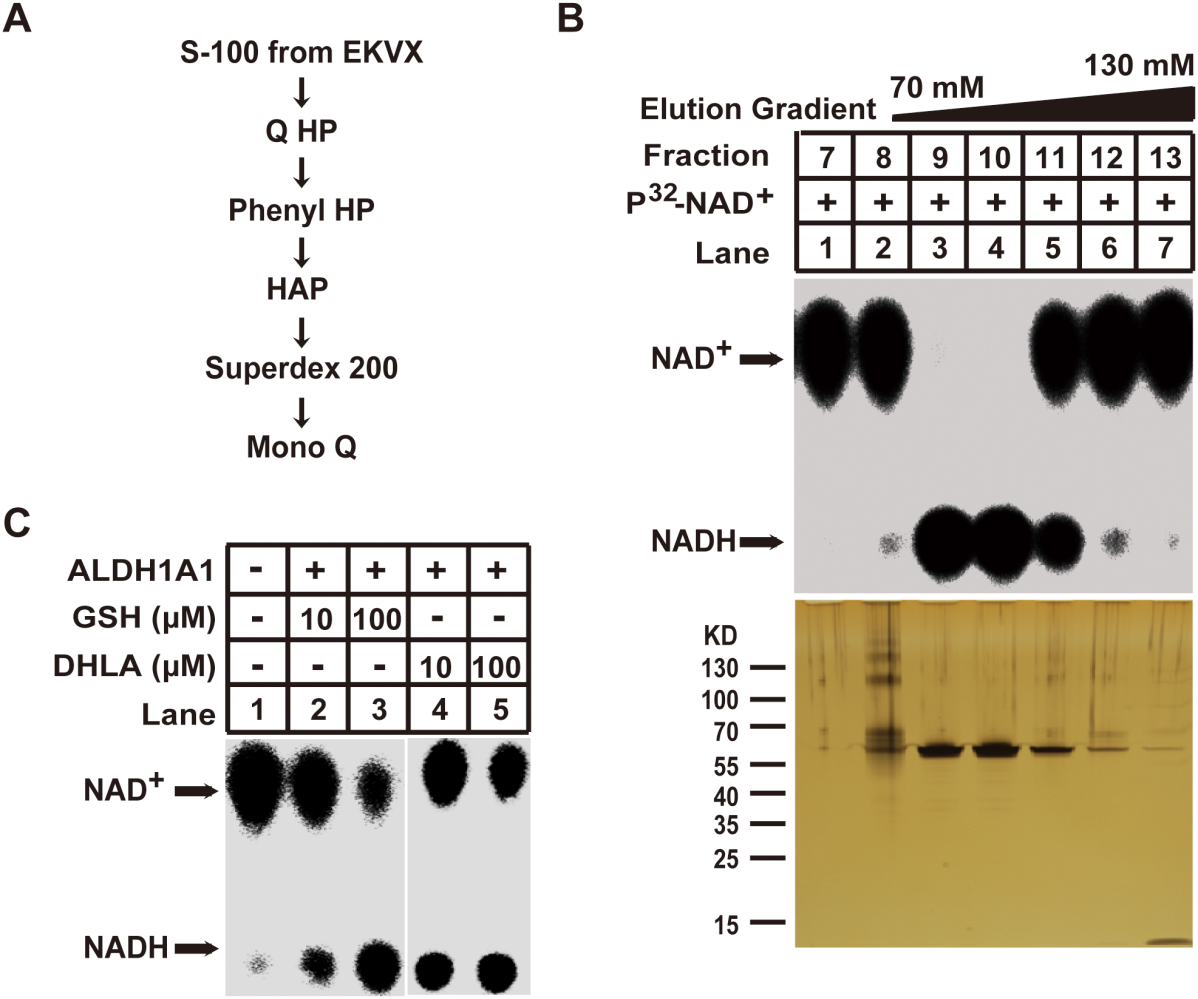
Purification of the GSH/DHLA-dependent NAD^+^-reduction activity **(A)** Diagram of the purification scheme for NAD^+^-reduction activity. **(B)** Final purification step. Top: 2 μL aliquots of the indicated fractions were assayed for NAD^+^-reduction activity as described in Figure 1A. Bottom: 20 μL aliquots of the indicated fractions were subjected to 4%–12% gradient SDS-PAGE gel followed by silver staining. **(C)** Recombinant ALDH1A1 protein was incubated with the indicated concentrations of GSH or DHLA in a total volume of 30 μl, and the NAD^+^-reduction activity was examined as described in the Materials and Methods.

We subsequently purified the enzyme(s) responsible for the GSH/DHLA-dependent NAD^+^ reduction activity from large-scale cell cultures. We separated 150 mg of S-100 cell extracts from EKVX cells via six sequential chromatographic steps as described (see Figure 2A and methods section). At the last purification step, a sharp peak of NAD^+^-reduction activity was eluted for fractions 9 to 11 (Figure 2B, upper panel). Subsequent silver staining revealed that one protein band at about 60 KDa is corresponding with the detected NAD^+^-reduction activity (Figure 2B, lower panel). This band was excised and analyzed via mass spectrometry. The protein was identified as aldehyde dehydrogenase 1 family member A1 (ALDH1A1).

To demonstrate that ALDH1A1 is responsible for the GSH/DHLA-dependent NAD^+^ reduction, we produced recombinant human ALDH1A1 (rALDH1A1) and tested it with *in vitro* assays. rALDH1A1 indeed reduced NAD^+^ to NADH in the presence of GSH or DHLA (Figure 2C).

### ALDH1A1 and ALDH3A1 have the same GSH/DHLA-dependent NAD^+^-reduction activity

**Figure 3:**
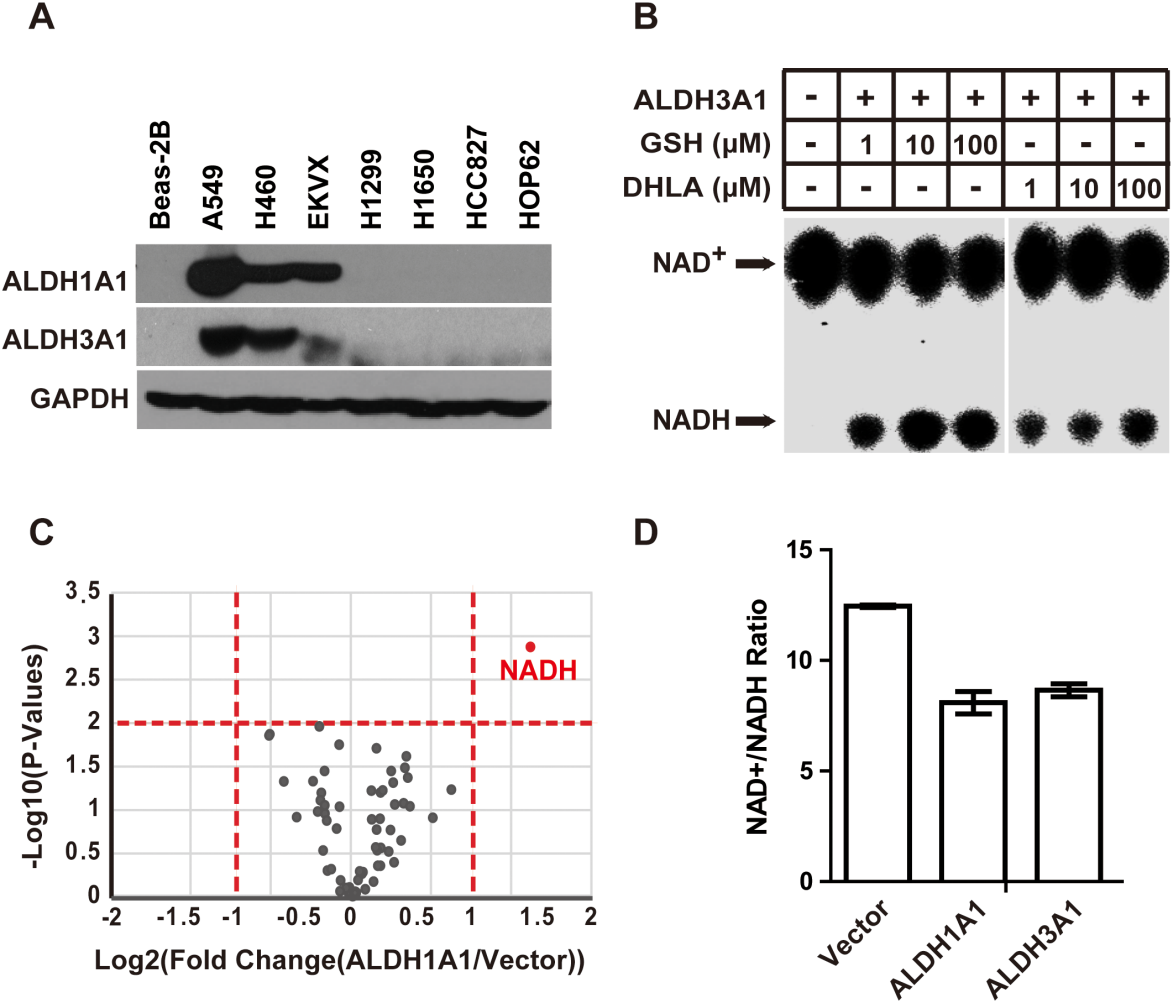
ALDH3A1 possesses the same GSH/DHLA-dependent NAD^+^-reduction activity as ALDH1A1 **(A)** Western blot analysis showing that the expression levels of ALDH1A1 and ALDH3A1 in the indicated lung cancer cell lines; GAPDH was used as the loading control. **(B)** Recombinant ALDH3A1 protein was incubated with the indicated concentrations of GSH or DHLA, and the NAD^+^-reduction activity was evaluated by TLC analysis. **(C)** Volcano plots showing changes in metabolite levels between ALDH1A1 overexpression cells and control cells. Metabolites of the indicated cells were extracted and subjected to targeted metabolite analysis as described in the Materials and Methods. Each dot represents one metabolite. The log fold change is represented on the x-axis. The y-axis shows the log10 of the *p* value. The red dashed lines represent a *p* value of 0.05 and a fold change value of 2. Metabolites (ALDH1A1 vs. Vector) with an accumulation fold change > 2 and a *p* value <0.05 are labeled as red dots. **(D)** Beas-2B cells were transfected with empty vector, ALDH1A1-expressing and ALDH3A1-expressing plasmids. Then, the NAD^+^/NADH ratios of the indicated cells were measured via triple quadrupole mass spectrometry. Each pooled sample was comprised of four biological replicates. Results are presented as the mean ± SD.

ALDH1A1 has been implicated in various pathologies, including cancer [21–24]. We therefore examined the expression of ALDH1A1 across multiple lung cancer cell lines. We found that ALDH1A1 is highly expressed in lung carcinoma cell line A549 and EKVX, as well as in the large cell carcinoma cell line H460 (Figure 3A and Supplementary Figure 2A). Another member of the aldehyde dehydrogenase family, ALDH3A1, showed a similar expression pattern with ALDH1A1 (Figure 3A and Supplementary Figure 2B). Other members of the aldehyde dehydrogenase family were not expressed in the lung cancer cell lines that we examined (data not shown).

To test whether ALDH3A1 has the same GSH/DHLA-dependent NAD^+^-reduction activity as ALDH1A1, we generated recombinant ALDH3A1 and assayed its NAD^+^-reduction activity in the presence of GSH and DHLA. ALDH3A1 efficiently catalyzed the reduction of NAD^+^ using GSH and DHLA as electron donors (Figure 3B), demonstrating that the GSH/DHLA-dependent NAD^+^-reduction activity is shared by ALDH1A1 and ALDH3A1.

To test whether ALDH1A1/3A1 can catalyze NAD^+^ reduction *in vivo*, we analyzed cellular metabolites following ALDH1A1 over-expression in Beas-2B cells using triple quadrupole liquid chromatography-mass spectrometry (TSQ LC-MS). Consistent with the *in vitro* activity, ALDH1A1 over-expression increased NADH levels. No other metabolites examined in the analysis changed significantly (Figure 3C, and Supplementary Table 1). Same as ALDH1A1, ALDH3A1 over-expression also decreased the NAD^+^/NADH ratio (Figure 3D).

### ALDH1A1 catalyzes the oxidation of aldehyde and GSH/DHLA via distinct enzymatic mechanisms

**Figure 4:**
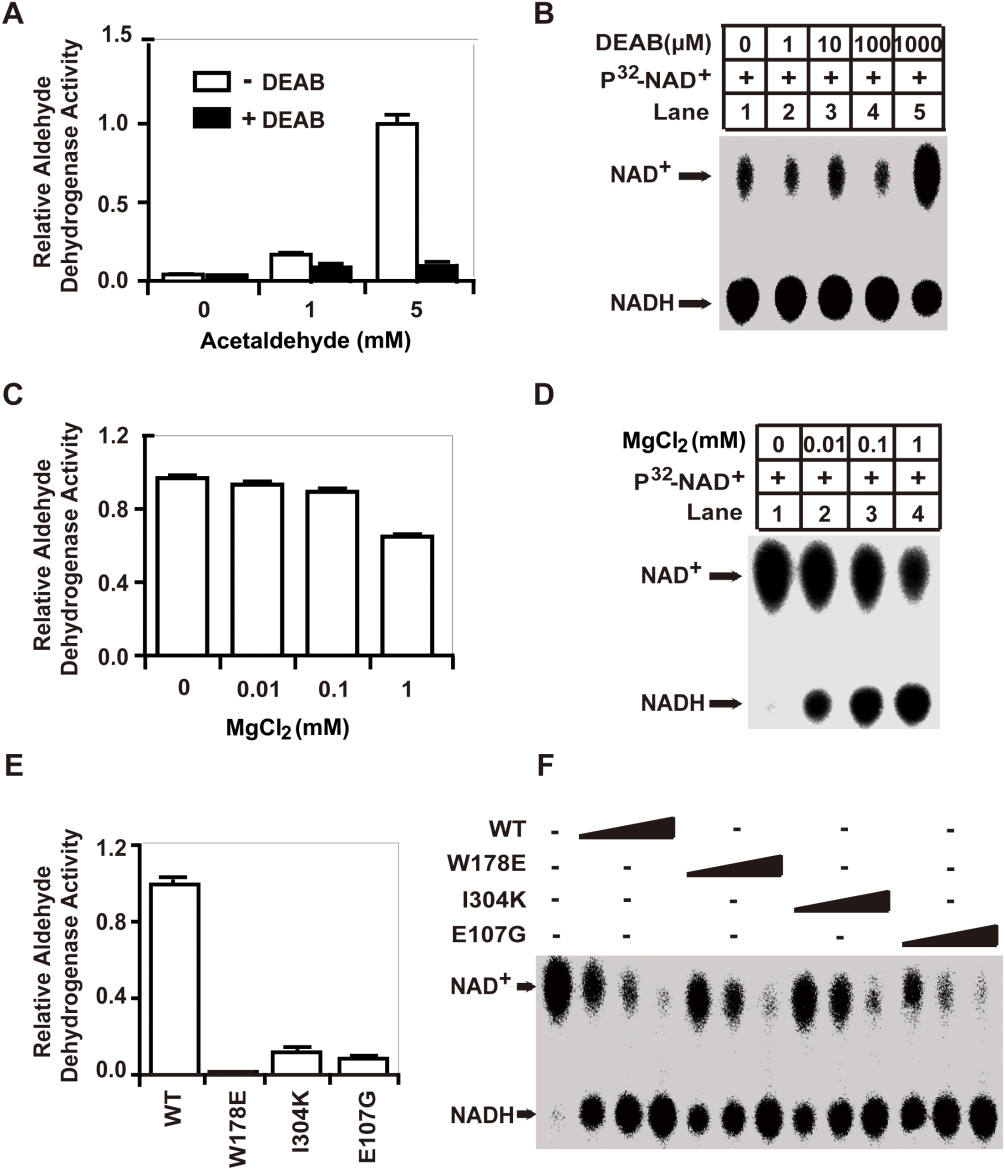
The enzymatic mechanisms of the aldehyde-dehydrogenase activity and the GSH/DHLA-dependent NAD^+^-reduction activity of ALDH1A1 are distinct **(A)** Aldehyde-dehydrogenase activity was assayed as described in the Materials and Methods. Briefly, recombinant ALDH1A1 protein was incubated with 1 mM NAD^+^ and different concentrations of acetaldehyde as indicated. NADH production was determined by measuring the absorbance at 340 nm. Aldehyde-dehydrogenase activity was assayed in the presence or absence of 10 μM of the ALDH1A1 inhibitor DEAB. **(B)** The GSH/DHLA-dependent NAD^+^-reduction activity was assayed in the presence of different concentrations of DEAB. (**C** and **D**) The aldehyde-dehydrogenase activity **(C)** and the GSH/DHLA-dependent NAD^+^-reduction activity **(D)** of ALDH1A1 were assayed in the presence of different concentrations of MgCl_2_. Results are presented as the mean ± SD. (**E** and **F**) The enzymatic activities of substrate-binding pocket mutants (W178E and I304K) and the glutamate 107 mutant (E107G) of ALDH1A1. **(E)** Aldehyde-dehydrogenase activity. **(F)** GSH/DHLA-dependent NAD^+^-reduction activity assays.

Previous studies have demonstrated that ALDH1A1 catalyzes the oxidization of various aldehydes to their respective acids in reactions that are coupled to the reduction of NAD^+^ to NADH [5, 9]. However, the NAD^+^-reduction activity we identified for ALDH1A1 in our assays does not involve aldehydes. Rather, this activity is GSH/DHLA-dependent. To further clarify this point, we used the well-characterized ALDH1A1 inhibitor diethylaminobenzaldehyde (DEAB), which competitively binds to the aldehyde-binding pocket of ALDH1A1, to inhibit aldehyde oxidation [25]. We found that 10 μM DEAB efficiently inhibited the aldehyde-dehydrogenase activity of ALDH1A1 (Figure 4A). However, the presence of DEAB, even at a 100 μM concentration, had no effect on the GSH/DHLA-dependent NAD^+^-reduction activity (Figure 4B). These results reinforce our initial idea that the GSH/DHLA-dependent NAD^+^-reduction activity is distinct from the previously-characterized aldehyde-dehydrogenase activity of ALDH1A1.

It has been reported that Mg^2+^ inhibits the aldehyde dehydrogenase activity of ALDH1A1 by inhibiting the dissociation of NADH from the enzyme; this dissociation is the rate-limiting step for ALDH1A1-mediated aldehyde oxidation [26]. Consistently, we found that 1 mM MgCl_2_ significantly inhibited the aldehyde-dehydrogenase activity. However, MgCl_2_ actually promoted the GSH/DHLA-dependent NAD^+^-reduction activity, and did so in a dose-dependent manner (Figure 4C and 4D). These findings suggest that Mg^2+^ exerts separate effects on the GSH/DHLA-dependent NAD^+^-reduction activity and the aldehyde-dehydrogenase activity of ALDH1A1.

To further investigate the enzymatic mechanism of the GSH/DHLA-dependent NAD^+^-reduction activity, we performed mutagenesis analysis based on the structure of ALDH1A1 [27]. When NAD^+^-binding sites (G246A/G251A) were mutated, ALDH1A1 lost both the aldehyde-dehydrogenase activity and the GSH/DHLA-dependent NAD^+^-reduction activity. When catalytic active center residues (C302A and C303A) were mutated, ALDH1A1 also lost both activities. Mutation of a cysteine residue outside the active center (C456A) had no effect on either of the enzymatic activities (Supplementary Figure 3A and 3B). However, when the aldehyde-binding pocket (W178E and I304K) and a residue that contributes to conformational integrity (E107G) were mutated, these mutants lost their aldehyde-dehydrogenase activity, yet completely retained their GSH/DHLA-dependent NAD^+^-reduction activity (Figure 4E and 4F, Supplementary Figure 3C and 3D). These results suggest that, even though they share the same NAD^+^-binding sites and the same active sites, the enzymatic mechanism of the GSH/DHLA-dependent NAD^+^-reduction activity of ALDH1A1 is distinct from its aldehyde-dehydrogenase activity.

### The GSH/DHLA-dependent NAD^+^-reduction activity of ALDH1A1 decreased the NAD^+^/NADH ratio *in vivo*

**Figure 5:**
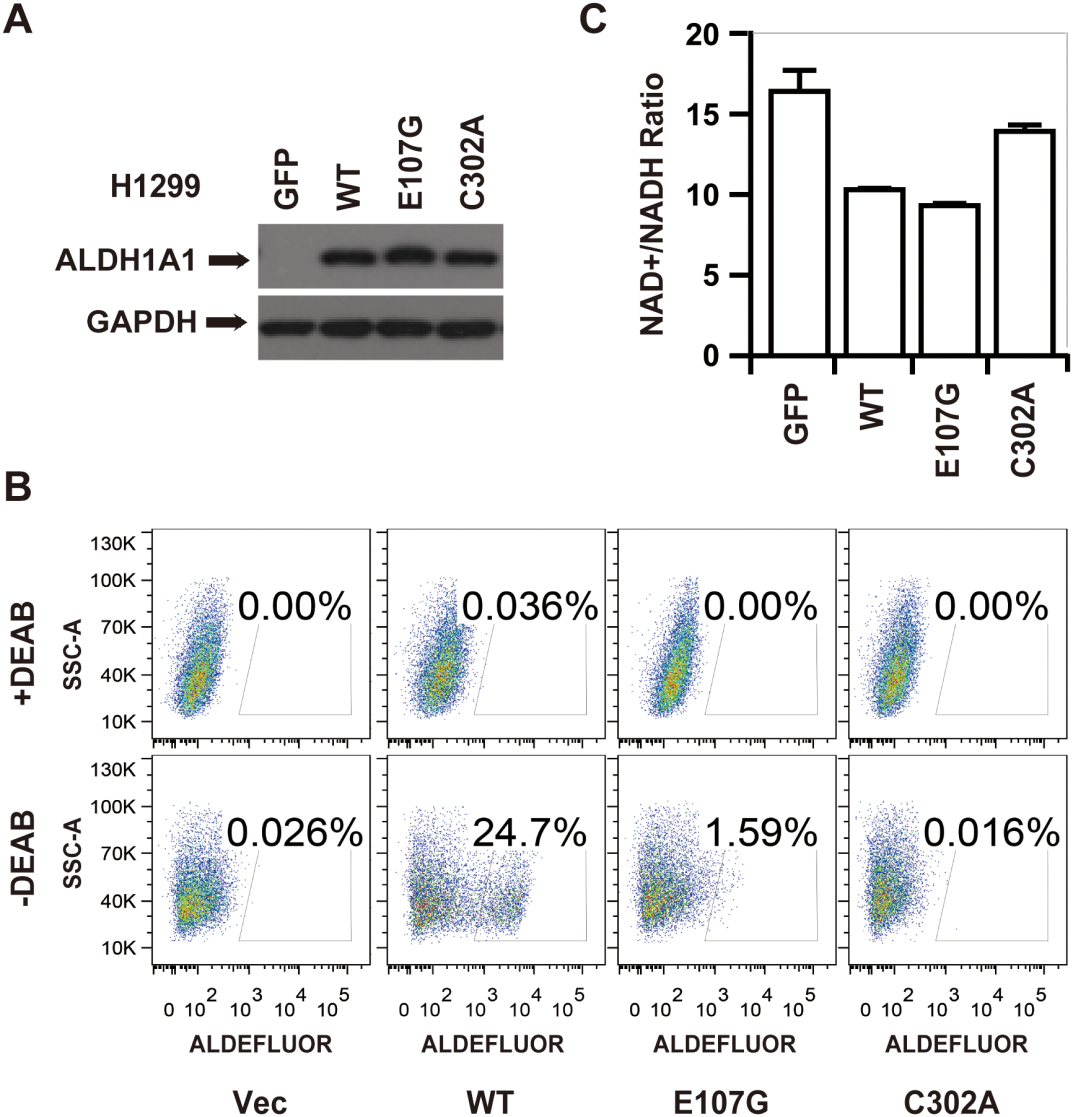
The GSH/DHLA-dependent NAD^+^-reduction activity caused NAD^+^/NADH ratio changes *in vivo* **(A)** H1299 cells stably expressing GFP or various forms of ALDH1A1 (wildtype (WT), E107G, or C302A) were generated following selection with puromycin for 5 days. The expression levels of ALDH1A1 and GAPDH were visualized by western blot analysis. **(B)** *In vivo* aldehyde dehydrogenase activity of the indicated H1299 stable cell lines visualized by ALDEFLUOR analysis. Cells incubated with the ALDEFLUOR substrate in the presence of the ALDH inhibitor DEAB were used to establish baseline fluorescence. Incubation of the indicated cells with the ALDEFLUOR substrate in the absence of DEAB induced a fluorescence shift indicative of aldehyde-dehydrogenase activity. **(C)** The NAD^+^/NADH ratios of the indicated stable cell lines were measured via triple quadrupole mass spectrometry. Each pooled sample was comprised of four biological replicates. Results are presented as the mean ± SD.

We established that overexpression of ALDH1A1 increased NADH levels and decreased the NAD^+^/NADH ratio (Figure 3D). To test whether the decrease in the NAD^+^/NADH ratio was caused by the GSH/DHLA-dependent NAD^+^-reduction activity of ALDH1A1 that we characterized *in vitro*, we ectopically expressed different mutants of ALDH1A1 in H1299 cells, a non-small cell lung cancer cell line that does not express endogenous ALDH1A1 (Figure 5A). Consistent with our findings from *in vitro* enzymatic assays (see Figure 4), H1299 cells expressing C302A, E107G, and I304K mutant forms of ALDH1A1 lacked aldehyde-dehydrogenase activity, as measured with an ALDEFULOR kit (Figure 5B and Supplementary Figure 4A). However, H1299 cells expressing the E107G and I304K mutant forms of ALDH1A1 had a decreased NAD^+^ to NADH ratio, similar to cells expressing the wild type ALDH1A1. The NAD^+^ to NADH ratio in cells expressing the catalytically-dead C302A form of ALDH1A1 did not differ from control cells lacking ALDH1A1 expression (Figure 5C and Supplementary Figure 4B). These results indicate that ALDH1A1 can affect the cellular NAD^+^ to NADH ratio via its GSH/DHLA-dependent NAD^+^-reduction activity.

### The GSH/DHLA-dependent NAD^+^-reduction activity of ALDH1A1 promotes tumor growth

**Figure 6:**
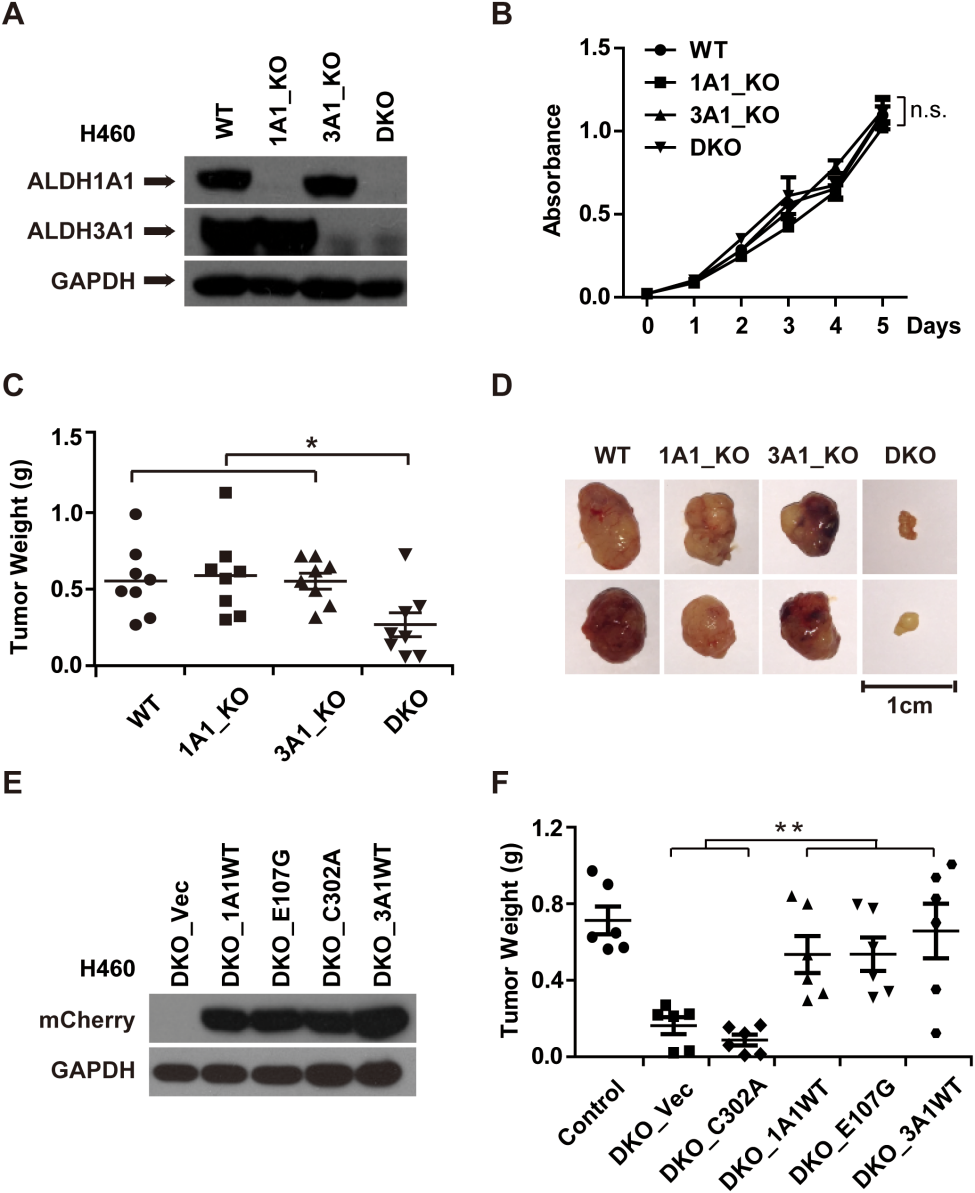
The GSH/DHLA-dependent NAD^+^-reduction activity of ALDH1A1 promotes tumor growth **(A)** ALDH1A1 knockout (1A1_KO), ALDH3A1 knockout (3A1_KO), and ALDH1A1/3A1 double knockout (DKO) H460 cell lines were established. The expression levels of ALDH1A1 and ALDH3A1 of the indicated cell lines were analyzed by western blot analysis; GAPDH was used as a loading control. **(B)** 10,000 cells/well of the indicated cell lines were seeded in 96-well plates on day 0. Cell numbers were determined with CCK-8 kit at the indicated time points after seeding. **(C)** Cells of the indicated cell lines (5 × 10^6^ cells/mouse) were implanted subcutaneously in the flanks of nude mice. After 27 days, mice were sacrificed, and tumors were excised. The weight of indicated tumors was determined. n = 8. *p* = 0.024. **(D)** Representative pictures of the excised tumors from each group are shown. **(E)** H460 DKO cells were rescued with empty vector (DKO_Vec), wildtype ALDH1A1 (DKO_1A1WT), two mutant forms of ALDH1A1 (DKO_E107G, DKO_C302A), and wildtype ALDH3A1 (DKO_3A1WT). The expression levels of the indicated proteins were measured by western blotting. **(F)** Cells of the indicated rescue cell lines (5 × 10^6^ cells/mouse) were implanted in the flanks of nude mice. Tumor weight was measured after the mice were sacrificed 27 days post implantation. n = 6. *p* = 0.006. *p < 0.05, **p < 0.01, n.s, not significant.

To evaluate the potential function of ALDH1A1 and ALDH3A1 in cell proliferation and tumor growth, we knocked out ALDH1A1 and ALDH3A1, both together (DKO) and individually (1A1_KO or 3A1_KO) (Figure 6A). The proliferation rates of 1A1_KO, 3A1_KO and DKO cells were similar to that of control cells (Figure 6B). However, when we measured tumor growth of these cells with deleted ALDH1A1 and/or ALDH3A1, we found that knocking out either gene alone did not inhibit tumor growth. However, the double knockout of these genes dramatically inhibited tumor growth (Figure 6C and 6D, and Supplementary Figure 5A). Re-introducing either ALDH1A1 or ALDH3A1 into cells restored the tumor growth in xenografts (Figure 6E and 6F, and Supplementary Figure 5B and 5C).

Having established that both ALDH1A1 and ALDH3A1 possess the GSH/DHLA-dependent NAD^+^-reduction activity, we tested whether or not this activity is responsible for the tumor-promoting effects observed for ALDH1A1/3A1. We took advantage of the mutants characterized in the experiments presented in Figure 4, and examined their potential rescue effects in the ALDH1A1/3A1 double knockout xenograft model. Of note, the E107G mutant form of ALDH1A1 restored tumor growth to the same extent as the wild type ALDH1A1. This restoration effect was not observed for the catalytically-dead C302A mutant form of ALDH1A1 (Figure 6E and 6F, and Supplementary Figure 5B and 5C). These results indicate that the GSH/DHLA-dependent NAD^+^-reduction activity of ALDH1A1/3A1 is responsible for the observed tumor promoting activity.

As have demonstrated that ALDH1A1 can decrease the cellular NAD^+^/NADH ratio, we wondered whether ALDH1A1 promotes tumor growth via decreasing the NAD^+^/NADH ratio. We measured the NAD^+^/NADH ratio in ALDH1A1/3A1 knockout cells. ALDH1A1_KO and ALDH3A1_KO cells had increased NAD^+^/NADH ratios compared with control cells, and the ratio was further increased in DKO cells (Supplementary Figure 5D). This result is consistent with the decreased tumor growth observed in the DKO cells. To further test if artificially increasing the NAD^+^/NADH ratio is sufficient to inhibit tumor growth, we expressed an NADH oxidase from *Lactobacillus brevis* (*Lb*NOX) in DKO_1A1 (ALDH1A1/3A1 double knockout rescued with ALDH1A1) cells [18]. As expected, expression of *Lb*NOX efficiently increased the NAD^+^/NADH ratio, and also inhibited the tumor growth in xenograft models (Supplementary Figure 5E and 5F). These results further demonstrate that the tumor-promoting effect of ALDH1A1 is, at least partially, through decreasing the cellular NAD^+^/NADH ratio.

## DISCUSSION

Aldehyde dehydrogenases are a group of NAD(P)^+^-dependent enzymes that catalyze the oxidation of aldehyde substrates. We found here that ALDH1A1 and ALDH3A1 have an NAD^+^-reduction activity that is independent of aldehyde oxidation. This activity is responsible for decreasing the cellular NAD^+^/NADH ratio and for promoting tumor growth in xenografts.

GSH and DHLA are potent antioxidants. They can effectively quench radicals and protect the thiol groups on proteins from oxidative damage [28]. They can also exert an antioxidative function indirectly, through regenerating other antioxidants such as vitamin C and vitamin E [29, 30]. Interestingly, we found that GSH and DHLA can regenerate another antioxidant, NADH, in a reaction catalyzed by ALDH1A1. This finding supports the idea that antioxidants interact with each other and function in a network [31]. In the future, structural analysis of ALDH1A1 in complex with GSH/DHLA will be needed to precisely characterize the catalytic mechanism.

The NAD^+^/NADH ratio has been considered to be a useful indicator of cellular metabolic status. Here, we found that the ectopic expression of ALDH1A1 decreased the NAD^+^/NADH ratio. This result is consistent with a recent report showing that ALDH1L1, another member of ALDH family, can decrease the cellular NAD^+^/NADH ratio and promote oxidative phosphorylation [32]. These findings suggest that revisiting the physiological functions of ALDH family members beyond their role in classic aldehyde oxidation reactions might yield new insights into the regulation of cellular metabolism.

ALDH1A1/3A1 are often over-expressed in cancers, but their function in cancer still remains unclear [33–35]. We showed that ALDH1A1/3A1 do not regulate cell proliferation. Instead, ALDH1A1/3A1 probably regulated processes including cell adhesion, vasculature development, and growth factor binding as suggested by our transcriptome profiling results (Supplementary Figure 6). Future studies explaining how ALDH1A1/3A1 regulate these downstream genes would help elucidate the exact function of ALDH1A1/3A1 in cancer. Several ALDH1A1 and ALDH3A1 inhibitors have been developed as potential anticancer treatments [36, 37]. Our results suggest that the inhibition of both ALDH1A1 and ALDH3A1, and particularly their reducing-agent-dependent NAD^+^ reduction activity, should be viewed as targets for the development of anti-cancer therapies.

## MATERIALS AND METHODS

### Plasmids and general reagents

Full-length human *ALDH1A1* and *ALDH3A1* were amplified and cloned into the pLVX-mCherry-N1 plasmid (Clontech) under the control of the cytomegalovirus (CMV) promoter. Point mutations were generated using a Quickchange Site-Directed Mutagenesis Kit (Stratagene) according to the manufacturer's instructions. *Lb*NOX gene was synthesized according to the provided sequence [18] and cloned into Lenti-blasticidin plasmid (Addgene). The following antibodies and reagents were used: anti-ALDH1A1 antibody (Abcam); anti-ALDH3A1 (Santa Cruz); anti-GAPDH antibody (Cell Signaling Technology); anti-mCherry antibody, which was a gift from Dr. Xiaocheng Wang's lab at NIBS.

### Cell culture and stable cell lines

Beas-2B and EKVX cells were cultured in Dulbecco's modified Eagle's medium (Invitrogen). H1299, A549, H460, Hcc827, H1650 and Hop62 cells were cultured in RPMI-1640 medium (Invitrogen). All media were supplemented with 10% Fetal Bovine Serum (Invitrogen) and 200 units/mL penicillin/streptomycin (Invitrogen). Cells were transfected using Lipofectamine 2000 (Invitrogen) according to the manufacturer's instructions. Lentiviral particles were generated by transfecting HEK 293T cells with PLVX and packaging plasmids. The Beas-2B and H1299 cell lines, stably expressing wildtype and mutant forms of ALDH1A1, were selected with 1 μg/mL puromycin after being infected with the corresponding lentiviruses. H460 cells stably expressing *Lb*NOX were selected with 10 ug/mL blasticidin after being infected with lentiviruses. *ALDH1A1* and *ALDH3A1* knockout H460 cells were generated using the CRISPR-Cas 9 system. *ALDH1A1* guide RNA sequences were as follows, gRNA-1, TGTCTTTAATCCTGCAACTG; gRNA-2, GCGAAAGAGATCGTCTGCTGC; gRNA-3, GTACTATGGATGCTTCCGAG. *ALDH3A1* guide RNA sequences were as follows, gRNA-1, AGAGGAGATCGAGTACATGAC; gRNA-2, GGAAGCTCCCTGAGTGGGCCG; gRNA-3, GGAAGACGCCCCAGACTCAGC.

### Preparation of S-100 cell extracts

Two days after seeding, cells were harvested at 90% confluency. After washing once with cold PBS, cell pellets were re-suspended in 5 volumes of ice-cold Buffer A (20 mM HEPES, 10 mM KCl, 1.5 mM MgCl_2_, 1 mM EDTA, 1 mM EGTA, 1 mM DTT, 0.1 mM PMSF, 0.1% CHAPS and complete protease inhibitor from Roche, pH 7.5) and incubated on ice for 30 min followed by homogenization. After centrifugation at 12,000 g for 10 min, the supernatants were further centrifuged at 100,000 g for 1 hour in a tabletop ultracentrifuge (Beckman). The resulting supernatant (S-100) was stored at −80°C until further use.

### Purification of ALDH1A1

150 mg S-100 extracts from EKVX cells were prepared as described above and applied onto a 10 mL Q Sepharose HP column (GE Healthcare) that had been freshly equilibrated with Buffer A. The column was eluted with a linear gradient from 50 mM NaCl to 300 mM NaCl. Fractions of 10 mL were collected, dialyzed overnight, and assayed for NAD^+^-reduction activity. The active fractions (30 mL) were pooled and heated at 65°C for 30 min. After centrifugation, ammonium sulfate was added directly to the supernatant to a final concentration of 200 mM. The mixture was equilibrated via rotation for 3 hour at 4°C, followed by centrifugation at 14,000 rpm for 30 min. The resulting supernatant was loaded onto a 5 mL Phenyl Sepharose column (GE Healthcare) equilibrated with Buffer A containing 200 mM ammonium sulfate. The column was eluted with a 100 mL linear gradient from Buffer A that contained 200 mM ammonium sulfate to Buffer A lacking ammonium sulfate. Fractions of 5 mL were collected, dialyzed overnight, and assayed as describe above. 20 mL active fractions were pooled, and were then loaded onto a 2 mL Hydroxyapatite (HAP) column (Bio-rad). The HAP column was then eluted with a linear gradient from Buffer A containing 25 mM phosphate to Buffer A containing 250 mM phosphate. Again, fractions were assayed, and active fractions were pooled and concentrated to 1 mL with a spin column (Millipore) and loaded onto a Superdex 200 (10/30) gel filtration column (GE Healthcare) equilibrated with Buffer A containing 100 mM NaCl. The column was eluted with the same buffer. Fractions were assayed, and a total of 3 mL of active fractions were pooled and loaded onto a 1 mL Mono Q column (Pharmacia). The column was washed with 10 column volumes of 70 mM NaCl in Buffer A and then eluted with a linear gradient from 70 to 130 mM NaCl. Fractions of 500 μL were collected and assayed for NAD^+^-reduction activity. Aliquots of 20 μL were subjected to SDS-PAGE followed by silver staining with a SilverQuest Silver Staining Kit (Invitrogen).

### Expression of recombinant proteins

cDNA for human *ALDH1A1*, *ALDH3A1*, *GAPDH*, *LDHA*, and *LDHB* were cloned into the pET28a (Novagen) vector with an N-terminal 6×His tag. BL21 (DE3) cells were transformed and grown at 37°C until the OD600 reached 0.6. Next, the bacterial culture was switched to 18°C and recombinant protein expression was induced by the addition of 0.5 mM isopropyl β-D-thiogalactoside (IPTG) and incubation overnight. Bacteria were harvested and then lysed by sonication. The recombinant protein was purified using Ni-NTA agarose beads (QIAGEN) following standard Ni-NTA purification protocol.

### *In vitro* assay for NAD^+^-reduction activity

Aliquots of cell extracts or purified enzymes were incubated with 3 μL 0.4 μCi/uL α-P^32^-NAD^+^ in a total volume of 30 μL of Buffer A at 30°C for 1 hour. The reactions were stopped by adding 3.5 μL 100% (W/V) trichoroacetic acid. After vortexing, the reaction mixtures were centrifuged for 10 min at 14,000 rpm at 4°C. Aliqutos of 1μL of the resulting supernatant were spotted on a TLC plate (Analtech, Cat# 105016) and air-dried. After being developed overnight in solvent (132 mL isobutyric acid, 36 mL ddH_2_O, and 6 mL of 30% ammonia hydroxide), the plate was air-dried and imagined by exposure to X-ray film at room temperature for 2 hour.

### *In vitro* assay for aldehyde-dehydrogenase activity

Recombinant ALDH1A1 protein (20 ng) was incubated with 1 mM NAD^+^ in sodium pyrophosphate buffer (10 mM, pH 7.5). Acetaldehyde at various concentrations were added into the mixture. NADH absorbance was measured at 340 nm once every five minutes for one hour.

### ALDEFLUOR assay

An ALDEFLUOR kit (STEMCELL Technologies) was used to quantify ALDH1 aldehyde-dehydrogenase activity *in vivo*. Briefly, 2×10^5^ cells were suspended in ALDEFLUOR assay buffer containing BODIPY-aminoacetaldehyde and incubated at 37°C for 40 min. For each sample, cell aliquots were incubated with or without diethylaminobenzaldehyde (DEAB), an ALDH-specific inhibitor. ALDEFLUOR fluorescence was detected using the FITC channel of fluorescence activated cell sorting (FACS). The ALDH-positive gates were established using the background fluorescence levels of the DEAB treated group.

### Metabolites extraction and targeted metabolic analysis

Cells were plated in 6-well plates and harvested two days later. The media was aspirated and cells were washed twice with cold PBS. Metabolites were extracted by adding an 80:20 methanol: water mixture immediately into the plate wells and incubating for 2 hour at −80°C. After centrifugation at 14,000 rpm, the resulting supernatants were lyophilized, resuspended in HPLC-grade water, and analyzed immediately to avoid NADH and NAPDH degradation. Targeted metabolic analysis was performed on a Thermo UltiMate 3000 Rapid Separation LC (RSLC) system coupled to a Thermo TSQ Quantiva triple quadrupole mass spectrometer. Student's *t*-tests were used to assess the statistical significance of differences between the control and experimental groups.

### Mouse xenografts

5×10^6^ H460 cells were inoculated into the lower flanks of 5-week-old female Balb/c nude mice. After an initial period of 10 days, the tumor volumes were measured every two days. Tumor volumes were calculated as follows: Volume = length × width^2^ × 0.5326. When the tumors reached 0.5-1 cm^3^. Mice were sacrificed and tumors were extracted and weighed.

### RNA-Seq and qRT-PCR

Total mRNA was extracted with TRIzol reagent (Invitrogen). RNA-Seq and data analysis were performed by Sequencing Center at NIBS. qRT-PCR were performed using SYBR Prexmix kit (Takara) on CFX96TM Real-Time PCR Detection System (Bio-Rad). The primers used are as follows,

ALDH1A1-F, 5’-GCACGCCAGACTTACCTGTC-3’;

ALDH1A1-R, 5’-CCACTCACTGAATCATG CCA-3’;

ALDH3A1-F, 5’-CAGTTACCGGGAGAGGCT GT-3’;

ALDH3A1-R, 5’-GTCCTGCCCGAGCTGAAG-3’;

VEGFA-F, 5’-TGGAATTGGATTCGCCATTT-3’;

VEGFA-R, 5’-TATGTGGGTGGGTGTGTCTAC AG-3’;

CP-F, 5’-TTGGGCCAATGAAAATATGC-3’;

CP-R, 5’-TCCAGGAGTAAACTCTCATTCTCA-3’;

SPP1-F, 5’-GTGATTTGCTTTTGCCTCCT-3’;

SPP1-R, 5’-GCCACAGCATCTGGGTATTT-3’;

ACTIN-F, 5’- GTCACCAACTGGGACGACA-3’;

ACTIN-R, 5’- TGGCCATCTCTTGCTCGAA-3’.

## SUPPLEMENTARY FIGURES AND TABLE


