# Supplementary material for: Aldehyde dehydrogenase 1A1 increases NADH levels and promotes tumor growth via glutathione/dihydrolipoic acid-dependent NAD+ reduction
Source: Oncotarget. 2017 May 8;8(40):67043–55. doi: 10.18632/oncotarget.17688 (PMC5620155; doi:10.18632/oncotarget.17688)
Supplement: Supplementary file 1 [file oncotarget-08-67043-s001.pdf]

Aldehyde dehydrogenase 1A1 increases NADH levels and promotes tumor growth via glutathione/dihydrolipoic acid-dependent NAD⁺ reduction

SUPPLEMENTARY MATERIALS

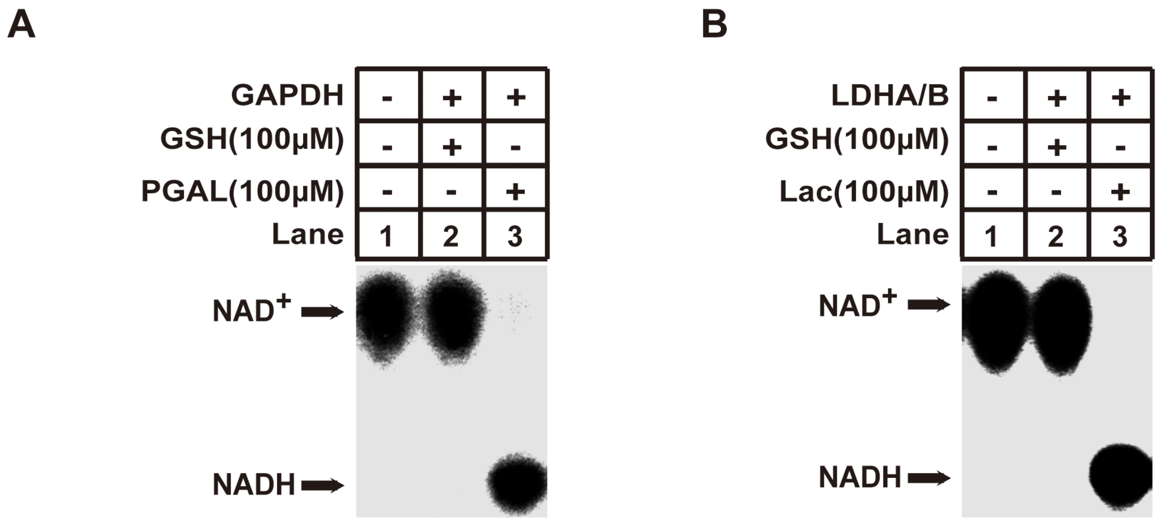

Supplementary Figure 1: The GSH/DHLA-dependent NAD^+-reduction activity is not shared by dehydrogenases GAPDH and LDHA/B. (A) Recombinant glyceraldehyde 3-phosphate dehydrogenase (GAPDH) was incubated with 100 µM GSH or glyceraldehyde 3-phosphate (PGAL) in the presence of P^{32}- NAD^+ for 1 hour, followed by TLC analysis. **(B)** Lactate dehydrogenases (recombinant LDHA and LDHB) were incubated with 100 µM GSH or lactate (Lac) at 30°C for 1 hour in the presence of P^{32}- NAD^+, followed by TLC analysis.

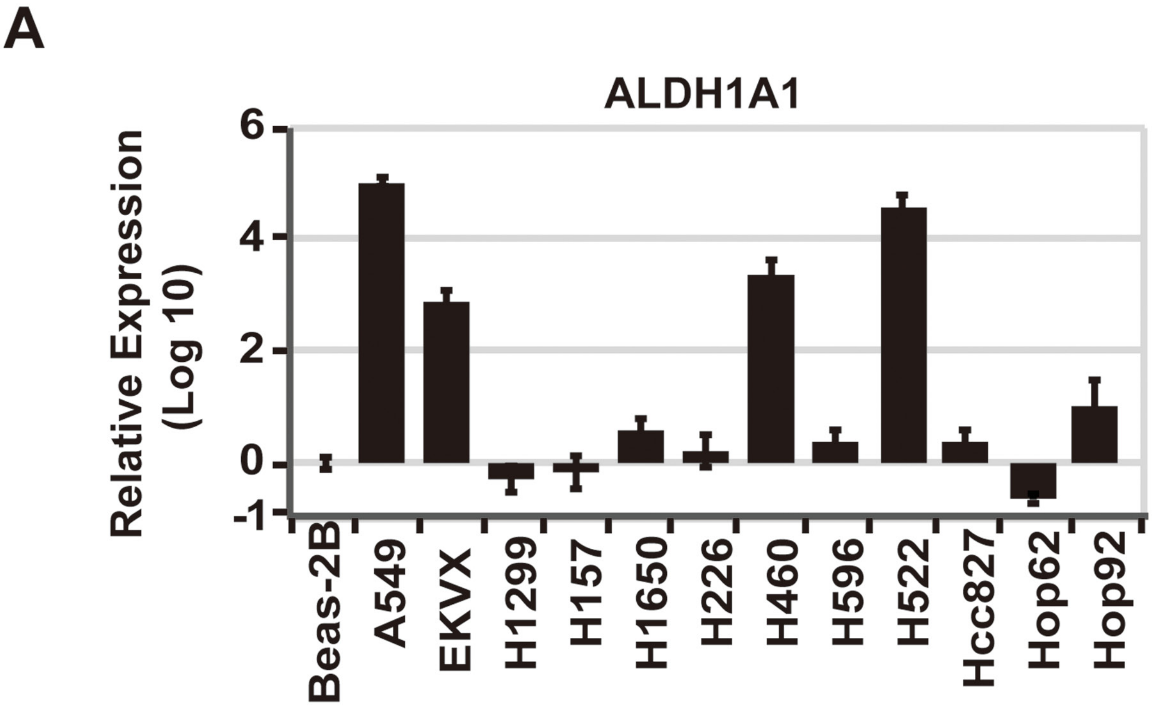

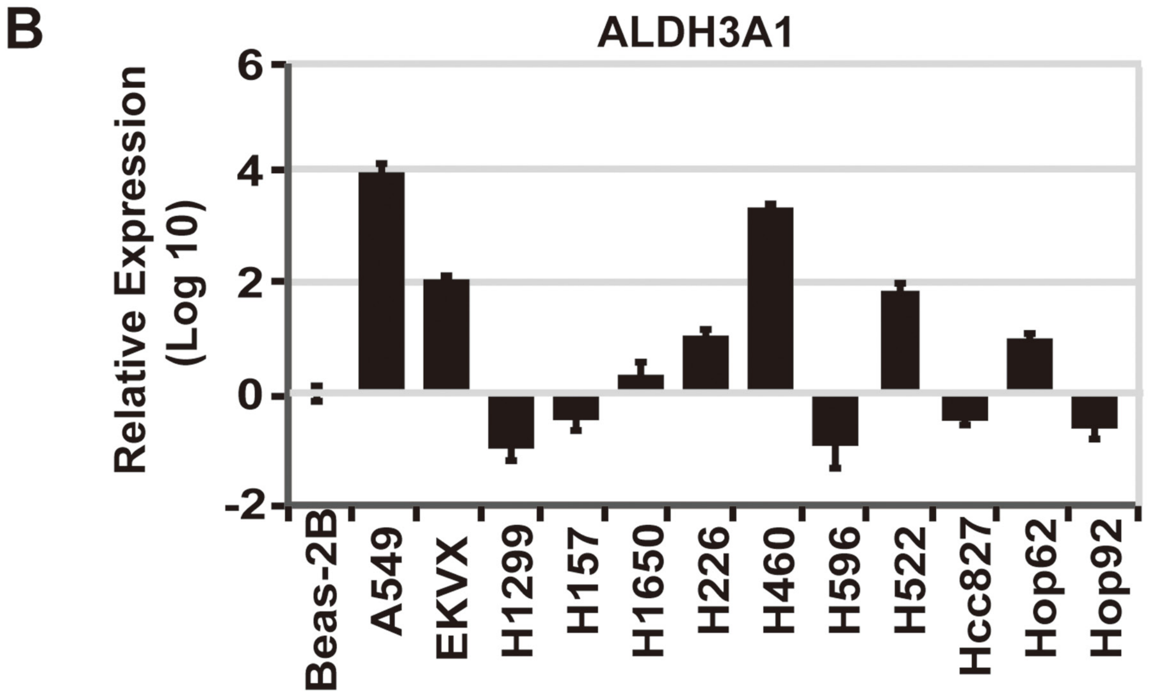

Supplementary Figure 2: Expression levels of *ALDH1A1* and *ALDH3A1* in different lung cancer cell lines. mRNA levels of *ALDH1A1* **(A)** and *ALDH3A1* **(B)** were quantified by q-PCR analysis (sample triplicates). The expression levels are normalized to that of *ACTIN*, and values are the relative expression levels compared with those in Beas-2B cells. Results are presented as mean ± SD.

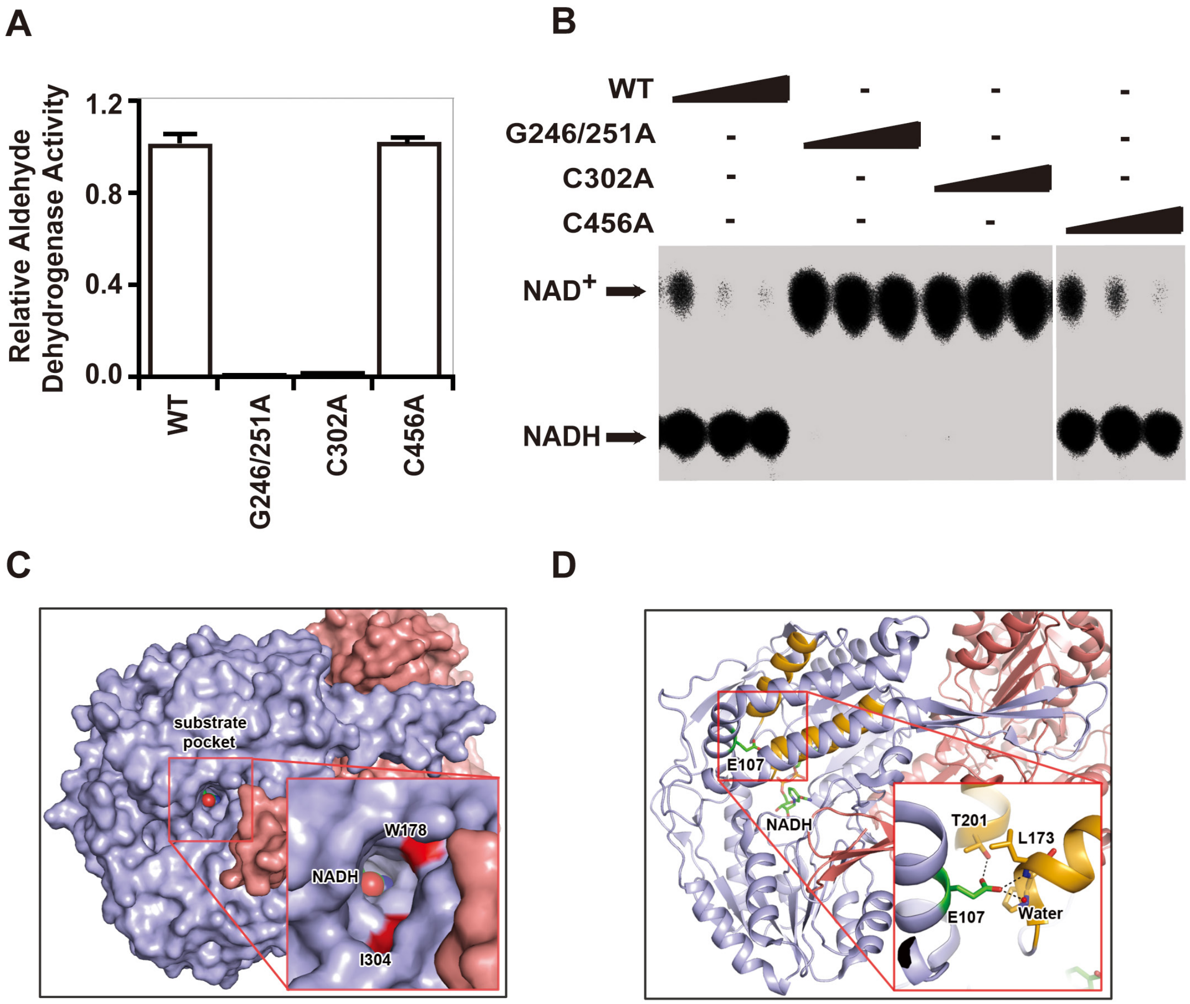

Supplementary Figure 3: Enzymatic activity of different mutant forms of ALDH1A1. (**A** and **B**) Determination of the enzymatic activities of NAD^+-binding site mutant (G246/251A), catalytic active site mutant (C302A), and cysteine 456 mutant (C456A). (A) Aldehyde-dehydrogenase activity assay. (B) GSH/DHLA-dependent NAD^+-reduction activity assay. **(C)** The surface structure of homodimeric human ALDH1A1. Two monomers are tinted in blue and salmon. Two hydrophobic residues (W178 and I304) in the substrate pocket are colored in red. **(D)** The structure of homodimeric human ALDH1A1 complexed with NADH (PDB: 4WB9) is shown. Two monomers are colored tint blue and salmon respectively. The side chain of E107 is shown; which is buried in the structure and engages in hydrogen bonds with residues from two helices (colored in orange).

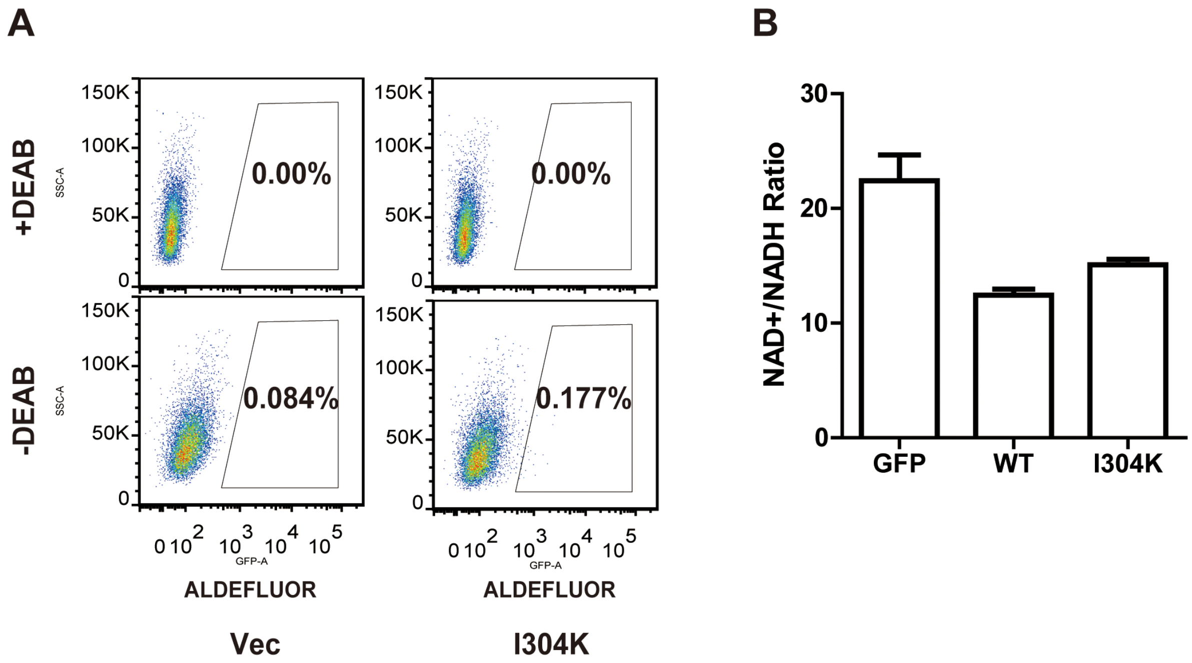

Supplementary Figure 4: Aldehyde binding pocket mutant of ALDH1A1 abolished aldehyde dehydrogenase activity while maintained the capability to decrease intracellular NAD$^+$/NADH ratio. **(A)** *In vivo* aldehyde dehydrogenase activity of the indicated H1299 stable cell lines was determined by ALDEFLUOR kit. **(B)** The NAD$^+$/NADH ratios were measured via triple quadrupole mass spectrometry. Results are presented as the mean ± SD.

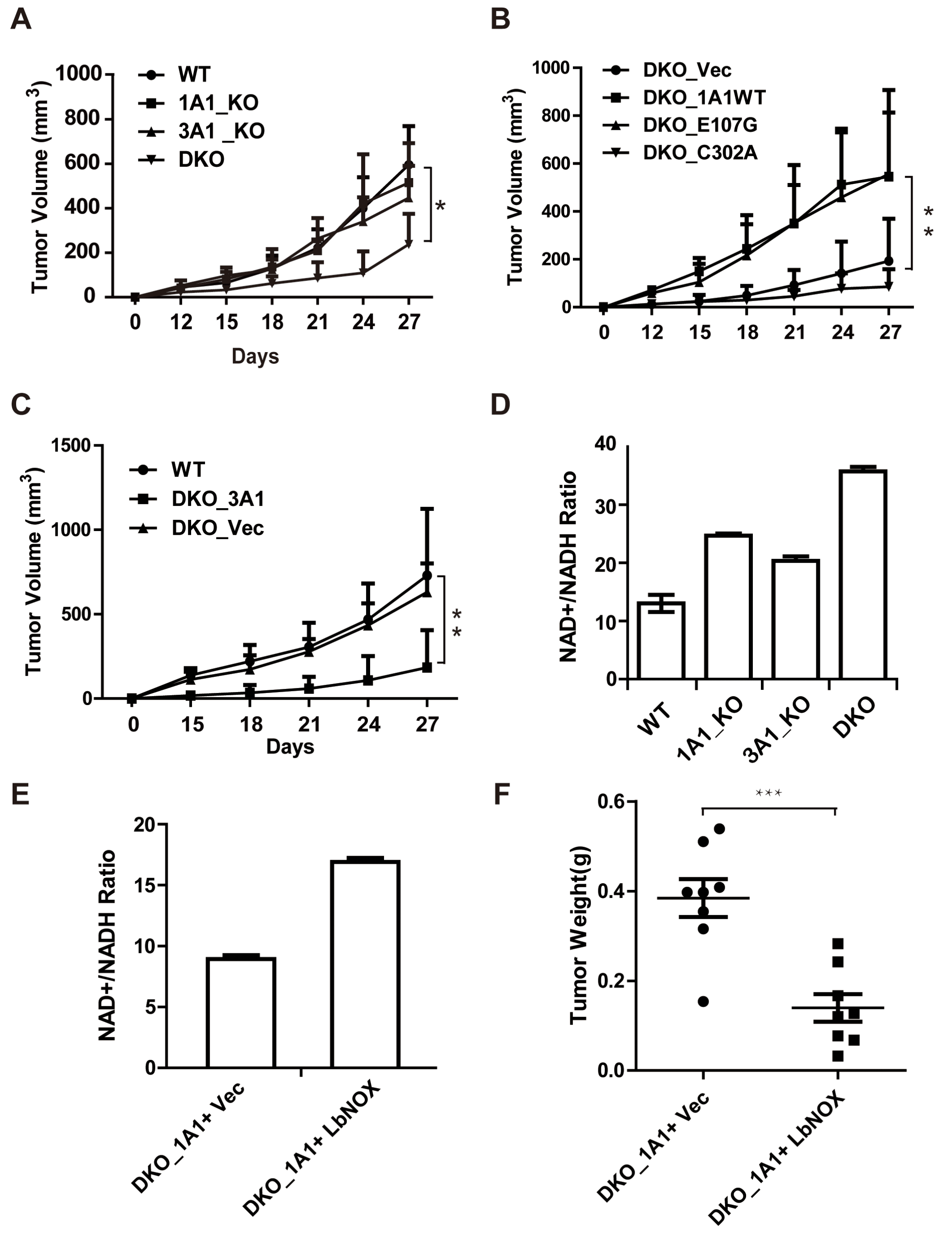

Supplementary Figure 5: ALDH1A1 promotes tumor growth via its GSH/DHLA dependent NAD$^+$ reduction. (A-C) Tumor volumes were measured using calipers at the indicated time points (days post implantation). Values represent the mean tumor volume ± SD. n = 8. **(D)** The NAD$^+$/NADH ratios of indicated H460 cells were determined by triple quadrupole mass spectrometry. Results are presented as the mean ± SD. **(E)** NADH oxidase from *Lactobacillus brevis* (*Lb*NOX) or empty vector were stably expressed in H460 DKO_1A1 cells (ALDH1A1/3A1 double knockout cells rescued with ALDH1A1). The NAD$^+$/NADH ratios of indicated cells were determined by triple quadrupole mass spectrometry. **(F)** Cells of the indicated cell lines (5 × 10^6 cells/mouse) were implanted subcutaneously in the flanks of nude mice. After 20 days, mice were sacrificed. Tumors were excised and weighed. n = 8. *$p < 0.05$, **$p < 0.01$, ***$p < 0.001$.

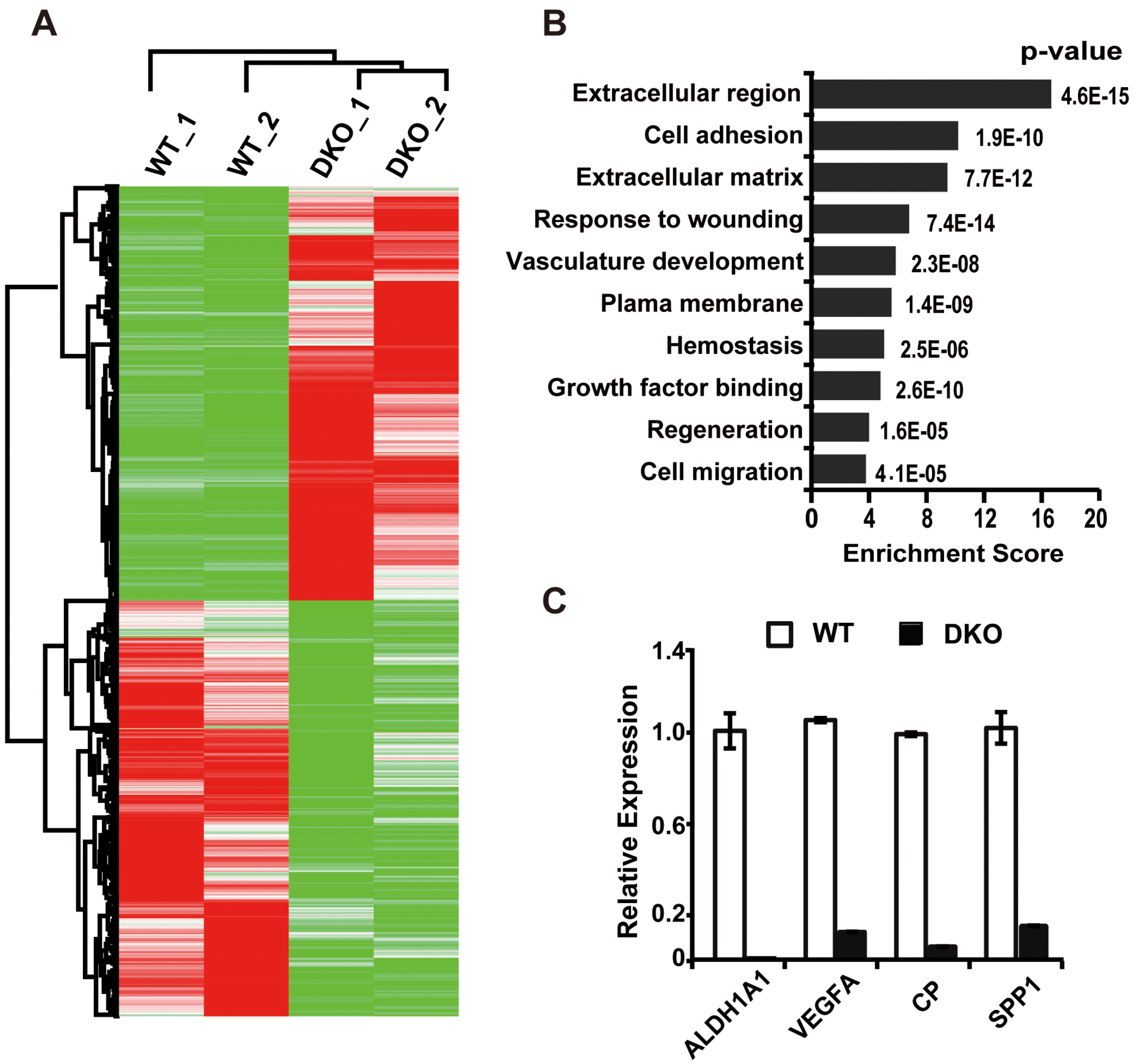

Supplementary Figure 6: Gene expression analysis by RNA-seq. (A) Heat map displaying 2198 differentially expressed genes between WT tumors and ALDH1A1/3A1 DKO tumors. (ANOVA adjusted *p*-value < 0.05). **(B)** Gene ontology analysis is performed with DAVID tools. (The enrichment p-values are corrected by Benjamini's methods) **(C)** mRNA levels of indicated genes in different tumors were quantified by q-PCR analysis (sample triplicates). The expression levels are normalized to that of *ACTIN*. VEGFA, vascular endothelial growth factor A, is a strong tumor angiogenesis inducer. CP, ceruloplasmin, is a major copper-carrier protein and promotes tumor angiogenesis in a copper-dependent manner. SPP1, secreted phosphoprotein 1, can facilitate cell adhesion to basement membranes and form an acidic microenvironment to promote tumor growth and invasion.

Supplementary Table 1: Metabolites were extracted from ALDH1A1 over-expression Beas-2B cells, or empty vector control cells, and subjected to targeted metabolic analysis. The peak abundance of individual metabolites was normalized, and listed in the table. Fold change and *p*-value were calculated.

See Supplementary File 1
